# Automated Ki-67 Quantification of Immunohistochemical Staining Image of Human Nasopharyngeal Carcinoma Xenografts

**DOI:** 10.1038/srep32127

**Published:** 2016-08-26

**Authors:** Peng Shi, Jing Zhong, Jinsheng Hong, Rongfang Huang, Kaijun Wang, Yunbin Chen

**Affiliations:** 1 School of Mathematics and Computer Science, Fujian Normal University, Fuzhou, Fujian 350117, China; 2 The Graduate School, Fujian Medical University, Fuzhou, Fujian 350004, China; 3 Department of Radiation Oncology, Laboratory of Radiation Biology, First Affiliated Hospital, Fujian Medical University, Fuzhou, Fujian 350005, China; 4 Department of Pathology, Fujian Provincial Cancer Hospital, Fuzhou, Fujian 350014, China; 5 Department of Radiology, Fujian Provincial Cancer Hospital, Fuzhou, Fujian 350014, China

## Abstract

Nasopharyngeal carcinoma is one of the malignant neoplasm with high incidence in China and south-east Asia. Ki-67 protein is strictly associated with cell proliferation and malignant degree. Cells with higher Ki-67 expression are always sensitive to chemotherapy and radiotherapy, the assessment of which is beneficial to NPC treatment. It is still challenging to automatically analyze immunohistochemical Ki-67 staining nasopharyngeal carcinoma images due to the uneven color distributions in different cell types. In order to solve the problem, an automated image processing pipeline based on clustering of local correlation features is proposed in this paper. Unlike traditional morphology-based methods, our algorithm segments cells by classifying image pixels on the basis of local pixel correlations from particularly selected color spaces, then characterizes cells with a set of grading criteria for the reference of pathological analysis. Experimental results showed high accuracy and robustness in nucleus segmentation despite image data variance. Quantitative indicators obtained in this essay provide a reliable evidence for the analysis of Ki-67 staining nasopharyngeal carcinoma microscopic images, which would be helpful in relevant histopathological researches.

Nasopharyngeal carcinoma (NPC) is one of the common cancers that occupies highest incidence rates in China and south-east Asia. Prognoses could be quite different even in NPC patients of the same clinical stage which are related to tumor-specific biological characteristics such as radiosensitivity and proliferation^1^. Most of NPC pathological patterns are non-keratinizing carcinoma and tumor cells outgrowth are active. Meanwhile, Ki-67 protein exists in the proliferation of cell nucleus, and the expression of which is strictly associated with cell proliferation and malignant degree^2^. Cells in the proliferation cycle which have higher Ki-67 expression (>10%) are always sensitive to chemotherapy and radiotherapy, and the treatment effect is better. Ki-67 expression can also be used to assess the prognosis of malignant tumors and evaluate the risk of distant metastasis. At present, many repots of Ki-67 were focused on breast cancer, lung cancer, gastric cancer, and colorectal cancer, which showed that Ki-67 expression in tumors after treatment and long-term effects of poor^3^. Clinical study showed that Ki-67 expression is closely related to the performance of NPC treatment, and patients with higher Ki-67 expression had better prognosis^4^. It is important to diagnose the malignant degree of tumor based on Ki-67 expressions, which makes IHC staining of Ki-67 an efficient tool for NPC cell characterization. Therefore, analyzing the microscopic images of Ki-67 staining tissue sections might provide an important evidence for NPC therapeutic assessment and prognosis.

In the microscopic image of a immunohistochemical (IHC) Ki-67 staining tissue section, positive cell nucleus is always stained by diaminobenzidine (DAB) and appear as brown, and negative cell nucleus is stained by hematoxylin as blue. Positive intensity of tumor cell is highly associated with the Ki-67 expression degree, which is also the depth of DAB shown in the image^5^. Besides, the morphological diversity of segmented nuclei is also important to understand proliferation activity of NPC cells. However, in most cases, staining deviation is unavoidable in the combination of pigments with Ki-67 protein. which brings difficulty in identifying differently stained cells. Manual Ki-67 assessment might have difficulties in distinguishing cell nucleus outlines and classifying cells due to the extremely uneven color distributions. Besides, manual quantification is also mind-numbing and time-consuming.

Researches on automatic cell nucleus segmentation of IHC staining images has been drawing on attentions recently, which save human labor and avoid subjective error in practice. Most of related researches are focusing on image segmentation methods based on thresholding, edge detection or machine learning based pixel classification. In which pixel intensity thresholding methods^6^,^7^ were to make use of pixel intensity in red, green and blue (RGB) color space, and applied intensity transformation and global thresholding according to differences between colors of brown and blue. Edge-based methods^8^,^9^,^10^ were to make use of pixel intensity, gradient flow or other characteristic morphological differences between both sides of the cell boundaries for segmentation, on which rely to look for boundaries. While classification methods took the single pixel as the object of study and pixels in the same category together constitute each component of tissues, in which both supervised^11^ and unsupervised^12^ learning approaches have been applied with the difference that whether training samples are needed. Researchers need to select representative area of each tissue components including all cell types as training samples before the supervised classification could be performed, the performance of which is highly affected by quality and comprehensiveness of pre-defined training samples. Besides, an imageJ^13^ plugin called ImmunoRatio based on color deconvolution^14^ was released to analyze Ki-67 images in an automated way, which also provided online quantification service for multiple immunostained tissue sections^15^. However, except for cell labeling illustrations, the only quantitative output is the ratio of DAB to nuclear area in that application. Therefore, a fully automatic Ki-67 assessment tool with multiple indicating outputs is highly needed in related pathological researches.

In practice, the color distributions in Ki-67 staining images are always extremely uneven, which makes nuclei have irregular and unclear boundaries. It is difficult for traditional image segmentation methods based on thresholding or morphological models to detect nucleus boundaries and quantify cells precisely. Taking single pixels as study objects, machine learning based methods classify pixels sharing similar characteristics as the same group, which perform the segmentation of Ki-67 staining image more efficiently, and then measurements derived from adjusted nucleus boundaries are provided for further pathological analysis after post-processing. The performance of machine learning based approaches are determined by two key components including high-relevant features and necessity of training samples, in which high-relevant features improve differentiation degree of pixels and scale of training sample selection affects efficiency and adaptability of the proposed method.

In this paper, we propose an automated image processing pipeline based on kmeans clustering without training samples needed, which is helpful for in-depth analysis of NPC tissue microstructures in clinic. The unsupervised image processing pipeline we proposed clustered pixels based on internal and external characteristics of pixels, in which both color intensities and local correlations were considered in image segmentation. Projected into the combined feature space, pixels were optimally separated into pre-defined tissue structures by growing clusters, in which DAB and hematoxylin nuclei separated from the background respectively. With morphological post-processing, precise boundaries of all segmented nuclei were obtained for quantitative analysis in pathological researches.

## Materials and Methods

**Figure 1:**
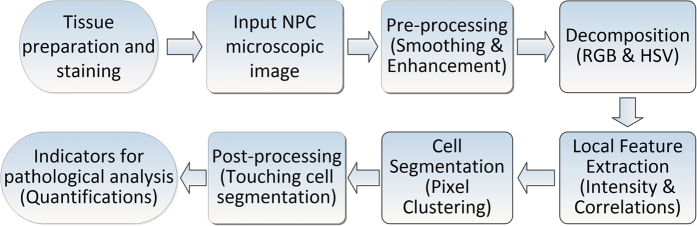
Workflow of proposed Ki-67 Staining NPC image processing and analysis pipeline.

To deal with color variability more efficiently and robustly as discussed above, a series of automated processes including image pre-processing, feature extraction, pixel clustering and touching nuclei segmentation are illustrated in Fig. 1. The distributions of Ki-67 expression can be extracted from the integrated workflow, which makes it possible to establish a whole set of indicator system for describing proliferation status of various cells.

All mouse procedures were approved by the Institutional Animal Care and Use Committees (IACUC) of Fujian Provincial Cancer Hospital and performed in accordance with institutional policies.

### Tissue preparation and image acquisition

Dataset preparation was performed on tissue samples of NPC from mice xenografts. Human nasoparyngeal carcinoma cell line CNE2 were provided by radiobiology lab, Fujian provincial cancer institute. Cells were cultured in RPMI-1640 media supplemented with 100 U/ml penicillin, streptomycin 100 μg/ml, 15% calf serum. Male BALB/c-nu nude mice aged from four weeks (Slack Laboratory Animal Co., Shanghai, China) were used for all experiments. For the primary tumor growth, 1 × 106 cells were injected into the right fore axillary region of the mice to form primary tumors. Mice were euthanatized when the maximum diameter of primary tumor reached 1.5 cm, and tumor tissue sections were fixed for pathological and IHC study. All tissue sections were dealt with Ki-67 staining, where general SP-9000 IHC kit (MXB Biotechnologies Co., Fuzhou, China) was adopted. Five complete and non-overlapping regions of interest (ROI) were randomly selected, and images were captured from an optical microscope with magnification factor of ×400 times, and stored as 2040 × 1536 × 24 bits jpeg files in size.

### Image pre-processing and decomposition

As the proposed clustering method was based on feature sets of single pixels, it was important to improve image quality before feature extraction. Usually Ki-67 staining images have two kinds of quality problems, which includes the uneven gathering of pigment on stained tissues, and small pigment particles scattering around. According to the features of the image, we have combined gaussian filter^16^ for smoothing and median filter^17^ for enhancement with five pixels in window radius together; meanwhile, most of the scatter noises were eliminated and local color intensity of pigment aggregates was enhanced.

### Local correlation feature set extraction

In order to segment different tissue structures from each other, single image pixels are treated as individual samples in the clustering and then aggregates of pixels sharing similar features form various nuclei, in which the definition of feature set is critical to determine distance between pixel samples in the feature space. Features adopted in the proposed method generally include three types as follows.

**Figure 2:**
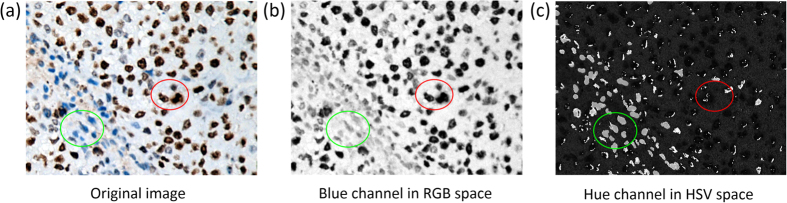
Illustration of signals decomposed from original images. (**a**) A zoom-in original image, (**b**) is the signal intensity map in blue channel of RGB space, and (**c**) is the signal intensity map in hue channel of HSV space respectively, where the red circle includes an area with brown nuclei, and the green circle includes blue nuclei in each maps.

(1) Blue color intensity (B channel) in RGB color space and hue intensity (H channel) in hue, saturation and value (HSV) color space respectively. B and H color channels are selected because of the intrinsic characteristics of Ki-67 staining image, where decomposed values of brown and blue have biggest differences comparing to other channels. As shown in Table 1, compared with other channels, color intensity of brown are 0 in both B and H channels, while blue has much higher value of 255 and 240 respectively. Those differences generate high relevant features for classification, which efficiently separate brown DAB nuclei, blue hematoxylin nuclei and the background from each other. As illustrated in Fig. 2, signals of brown DAB nuclei have much higher contrasts in the decomposed blue color channel, and blue hematoxylin nuclei are easily identified in hue color channel rather than in original images, which would separate three types of pixels more efficiently in clustering than taking all signal channels into account. The high relevant feature set based on B and H channels represents pixels well scattered in the high-dimensional feature space for the following clustering.

(2) Mean (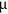) and standard deviation (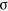) value of a single pixel and its 3 × 3 neighborhood pixels in B and H color channel respectively, in which 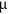 shows mean signal strength and 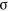 shows signal variance in the local area, where x_i_ is the signal intensity of a pixel in the local 3 × 3 window.


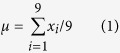



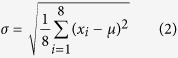


(3) Local texture features including skewness (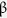) and kurtosis (β_k_) of a single pixel and its 3 × 3 neighborhood pixels in B and H color channels respectively, in which 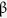 measures symmetry of local signals, and β_k_ measures whether the signal intensities are peaked or flat relative to a normal distribution.


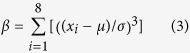



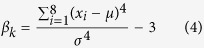


Correlations of neighborhood pixels are considered in feature selection, which help clustering local pixels with similar color and intensity, and separating neighborhood pixels with big differences in color or intensity.

### Nucleus segmentation based on kmeans clustering

The basic idea of kmeans clustering method is to initially make k cluster centers given at random, k = 3 in this paper, and calculate the barycenter of each sample cluster into new cluster center and iterate until the displacement distance of cluster center is less than a given value. Despite variances of features including color intensity and neighborhood correlations, it was specified to segment pixel sample set into DAB nuclei, hematoxylin nuclei and background in Ki-67 staining NPC images.

**Figure 3:**
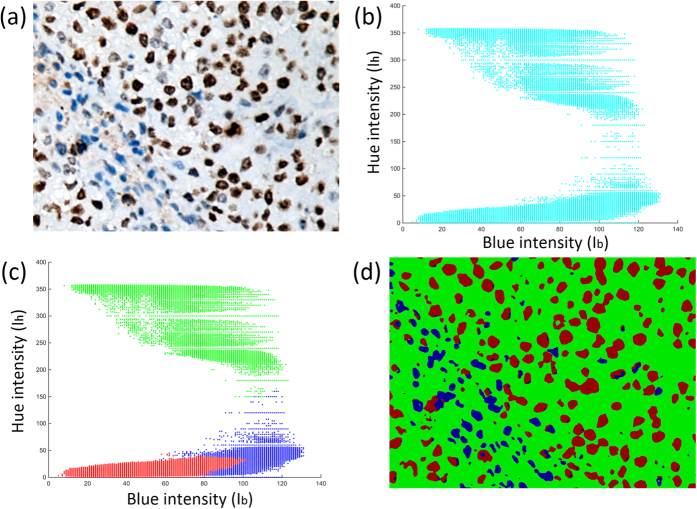
Illustrations of nucleus segmentation based on kmeans clustering. (**a**) An original sample Ki-67 staining NPC image, (**b**) Distribution map of all pixels before clustering in the feature space, (**c**) Distribution map of pixels after clustering, where red circles represent as pixels of DAB nuclei; green circles represent as background and blue circles represent as hematoxylin nuclei; the coordinate axises represent as blue and hue intensities respectively of each pixel, (**d**) Color map of labeled NPC image pixels after kmeans clustering corresponding to (**c**), where red represents as DAB nuclei, green as background and blue as hematoxylin nuclei.

Generally there is a ten-dimensional vector including (I_b_, I_h_, μ_b_, μ_h_, σ_b_, σ_h_, β_b_, β_h_, β_kb_, β_kh_) projected into the multiple feature space, and only intensities of blue and hue channels (I_b_ and I_h_) are selected to illustrate pixel distributions in a two-dimensional plane. Three clusters are well separated as shown in Fig. 3, representing the accuracy of the unsupervised clustering based on the proposed features.

### Touching nuclei segmentation based on watershed

In histopathological study, it is often required to analyze different cell structures in the NPC tissues such as nucleus counting, nucleus classification and other corresponding measuring indicators. Therefore, further segmentation is required to find the accurate boundaries of each nucleus. However, in practice, post-processing is needed to adjust segmented nucleus shapes including erasing too small regions, filling holes and gaps, and most of all, separating adjacent nuclei due to staining problems, which are shown as the touching nuclei on the image, and bring errors to morphological measurement based on single cells.

A kmeans clustering based method was proposed by Al-Lahham *et al.*^18^ for proliferation rate estimation in breast cancer, in which pixels were clustered into various tissue structures fully automatically, but the overlapping problem has not been resolved satisfactorily using global thresholding, mathematical morphology and connected component analysis. As a widely used method in grey-scale image segmentation, Watershed has been applied in many researches to segment touching cells^19^,^20^. In watershed, a grey-scale image may be seen as a topographic relief, where the grey-scale level of a pixel is interpreted as its altitude in the relief. Gradient transformation was needed to separate attached nucleus from each other based on catchment basins formed by gradient changes between them. Starting from an initial grey-scale image, morphological gradients were calculated based on differences between the initial image and the gray-scale image, is to be used^21^ to form the gradient map. Taking chessboard distance map to edge of each closed region as an initial shape marker for watershed, where (x_1_, y_1_) and (x_2_, y_2_) are positions of the pixel p_1_ inside a nucleus and p_2_ on the edge respectively.


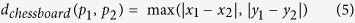


**Figure 4:**
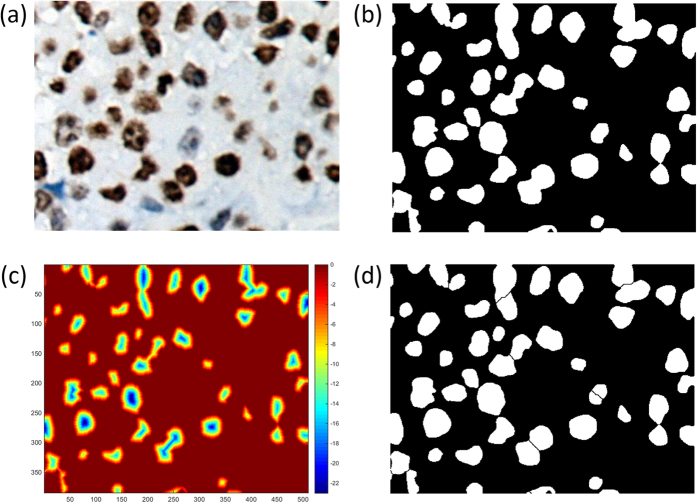
Steps of touching nuclei segmentation. (**a**) A zoom-in original image including touching nuclei, (**b**) bitwise map, (**c**) heat map of distances to closed region edges, where x and y axis are original image coordinates, and color bar shows the chessboard distance, and (**d**) Isolated cell nuclei with watershed boundaries.

Assuming that there exists one-to-one correspondence between the shape markers and the desired nucleus objects. Distributions of each nucleus for further measurements based on clear boundaries shown in Fig. 4, giving a comprehensive morphological view of nucleus for in-depth analysis.

**Ethics and consent statements.** All mouse procedures were approved by the Institutional Animal Care and Use Committees (IACUC) of Fujian Provincial Cancer Hospital and performed in accordance with institutional policies.

## Results

To validate our proposed algorithm, we use the dataset including 20 Ki-67 staining NPC tissues sections, and totally 100 microscopic images were captured from five randomly selected visual fields in each section. Our method was implemented in MATLAB and deployed on a PC with 3.0 GHz CPU and 8G RAM. In practice, it took 1.7 seconds in average for this algorithm to process one RGB image of 2040 × 1536 pixels in the preliminary test, which was fully competent to meet the real-time requirements in clinical use. The high speed and adaptability of this algorithm made it possible to be applied in the clinic biological research of large NPC tissue sample analysis, which saved manpower and avoided subjective errors.

### Stability conducted in Ki-67 staining NPC tissue image quantifications test

To validate the automated segmentation techniques against the manual efforts, nuclei counting by the proposed method was tested to evaluate the consistency of our algorithms. Only DAB nuclei representing true positive staining were labeled by two human experts as gold standards. We also used the ImmunoRatio software, an ImageJ plugin for analyzing images of estrogen receptor (ER), progesterone receptor (PR) and Ki-67 immunostained tissue sections, to detect and label nuclei for comparison. Except for comparison of the main features between two approaches shown in Table 2, we mainly compared the ratio of DAB to nuclear area between them, which is the only output of ImmunoRatio besides the result images.

From Table 2, a comprehensive view of similarities and differences between two automatic methods is presented. The main differences existing in nuclei labeling part make our algorithm more adaptable because of the unsupervised machine learning strategy. The overall background differences do not affect the correlations between local pixels so no background correction is needed. Low quality images are also acceptable due to the robustness of clustering method, and most of the clustering errors can be corrected in the morphological post-processing. Meanwhile, since ratio of DAB to nuclear number is the only output value of ImmunoStudio, we compare this measurement with the error rate against gold standard for quantitative evaluation.

**Figure 5:**
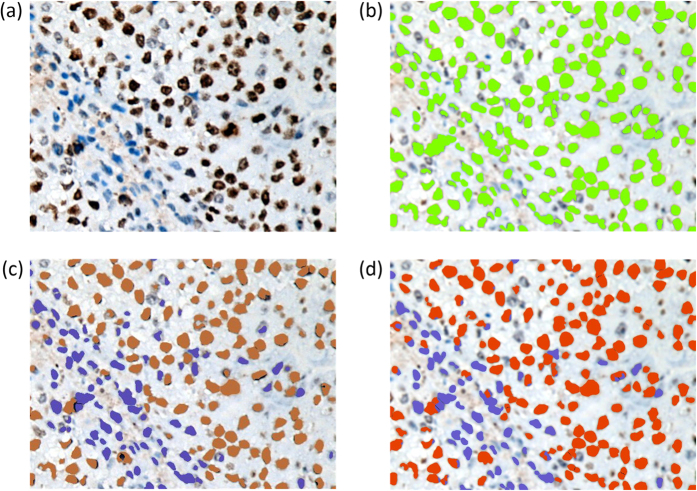
Comparison of nuclei segmentation results. (**a**) An original Ki-67 staining image, (**b**) all labeled cell nuclei against background marked as green, and results of isolated nucleus labeling using (**c**) ImmunoRatio, and (**d**) our method, where DAB nuclei are marked as orange, and hematoxylin nuclei are purple. Slight differences were made between marker colors in (**c**,**d**) for a better comparative view.

Both of two automatic methods have similar output values in DAB/nuclear number as listed in Table 2. The demo results shown in Fig. 5 also illustrate that similar nuclei labeling and overlapping segmentation were conducted by two methods, and differences could be found with focusing on details. Meanwhile, a little lower error rate of our method was obtained, which was mainly because of the following reasons.

First, most of the nuclei with very light colors were discarded and labeled as background by both of two algorithms, and some very small color aggregates in centers of those ghost nuclei were also discarded in our method. The main reason was that adjustable nucleus area threshold was set for filtering those tiny fake nuclei.

Second, less morphologically incomplete nuclei could be found in our result images, which was mainly because more local correlation features were considered in clustering, and those correlations helped neighborhood pixels with similar features clustered together.

Third, dealing with those nuclei with fuzzy mixed colors that were stained by both DAB and hematoxylin, our algorithm presented better classification accuracy because of the comprehensive feature set from two color spaces. Values in hue channel played an important role in these cases to separate different parts inside the same nuclei according to distances in the feature space.

Generally the proposed method outperformed well in segmentation of most touching nuclei correctly, while error rate of nucleus segmentation was composed of false accept rate (FAR = 5.5%) and false reject rate (FRR = 2.7%). In this case, FAR checking took those tiny fake nuclei and the over-segmented nuclei into account, which concentrated in the segmentation of touching nuclei and unreasonable boundaries were generated by watershed. Simultaneously, the composition of FRR included missing nuclei and the under-segmented nuclei, where the touching nuclei were not successfully separated. With the segmentation accuracy of 91.8%, results of proper nuclei labeling and segmentation built solid foundation for positive grading analysis of in histopathological researches.

In addition, appropriate sample size was required to be determined for statistical and reliability testing because of the limited tissue source. In the paired *t* test, the minimum number (N) of samples required by the experiment could be calculated following the equation below.


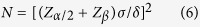


where *δ* is the allowable error which shows the overall mean difference, *σ*_*d*_ is the population standard deviation of the difference value, *Z*_*α*/2_ is the two-tailed critical value of the standard normal distribution, *Z*_*β*_ is taken into account regardless of one or two tailed test, and *α* and *β* are set as 0.05 and 0.1 respectively in this case.

After the presented preliminary experiment, the mean accuracy of 20 sample slides derived by the proposed method was 75.1 ± 6.7%, in which *σ*_*d*_ = 6.7. The allowable error *δ* was set as 5%, and *Z*_*α*/2_ = 1.96, *Z*_*β*_ = 1.282 as fixed numbers were derived respectively from the two-tailed test. Taking those parameters into equation of N, the minimum number of required by the experiment could be calculated as 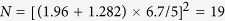, which showed that N = 20 is sufficient to prove extendibility of the proposed method in a larger scale dataset.

### Positive grading analysis of Ki-67 staining NPC tissue images

To evaluate Ki-67 expression of NPC tissue images in a comprehensive way, a grading indicator was applied in our experiment according to the classic method in ref. 22, in which the positive level of NPC cells was a critical indicator in IHC examines, and defined as the product of coloring strength and percentage of positive cells.

**Figure 6:**
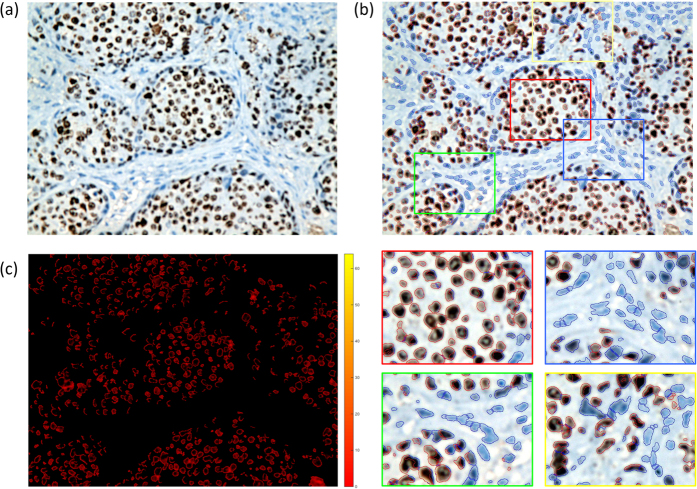
Illustration of Ki-67 expression assessment. (**a**) Original image of one tissue slide, (**b**) positive and negative cell detection and segmentation results (upper) and several zoom-in patches for better illustration (under), and (**c**) highlighted positive cells, where darker red inside cells represent higher Ki-67 expressions.

As shown in Fig. 6, number of both negative and positive cells could be drawn from each tissue slide after overlapping segmentation, and on the basis of which the calculation of Ki-67 expression could be performed. Four zoom-in regions include three typical types were selected for a better view, where the majority of upper left sub-graph are positive cells, the majority of upper right are negative, and numbers of positive and negative cells are almost equal in the under two sub-graphs, which fully reflect performance of the proposed method.

Taking each tissue slide into account, Ki-67 expression was classified into four grades based on the calculation of Ki-67 positive cells percentage in each section, where positive percentages of 0~5% were ranked as (−), 6~25% as (+), 26~50% as (++), and 50~100% as (+++), and the final grade was the average of all five visual fields in one section. According to criteria defined in ref. 23, (−) and (+) were low level expression, (++) was medium level expression and (+++) was high level expression. Statistical analysis were performed on 20 NPC tissue sections.

As shown in Table 3, quantitative DAB coloring strength could be derived by our method rather than manual detection. In order to acquire the general positive percentage of the dataset, manually and automatically detected DAB and all nuclear numbers were selected firstly from all five visual fields in each of the examined slides. Secondly, mean and standard deviation values of positive percentage in each slide were then calculated based on those detection results of those five visual fields. Then, after checking the consistency between manual and automated quantifications via paired *t* test with p value lager than 0.05, showing the conformity between manual and automated results, and finally the mean and standard deviation values of the general positive percentage were considered as the average of 20 slides.

Based on 100 Ki-67 staining images from 20 NPC tissue sections, generally the proposed method derived the same positive grading results as human experts did. All sections were correctly classified automatically with high accuracy and efficiency. Since we count positive nuclei based on DAB coloring strength rather than artificial judgment, value of I_b_ intensity was listed in the first row. The higher average DAB/nuclear number of our method was mainly because of less hematoxylin nuclei detected. Normal nuclei stained by hematoxylin were always smaller in size and had unclear boundaries against the background, which were easily to be discarded in automated segmentation and caused increase in statistics of positive percentage. Meanwhile, false accepted nuclei were another reason of higher positive percentage, in which more fake positive nuclei detected by our method than manual result. However, those errors were still under a reasonable level and could be reduced by further improvements of image processing algorithms.

## Discussions

In IHC examine of NPC, inspection of tumor proliferative activity is critical to estimate the positive degree, predict biological behavior and evaluate prognosis. Experimental results showed that the proposed clustering algorithm has close outcomes as manual quantification with more efficiency and the web-based automated image analysis application ImmunoRatio with more quantification outputs. In this paper, we propose a fully automatic Ki-67 staining image processing pipeline to analyze NPC tissue sections. After smoothing and enhancement of original microscopic image, pixels were clustered based on the local correlation features in multiple color spaces. DAB and hematoxylin nuclei were composed by labeled pixels and separated from the each other. Post-processing including morphological adjusted and touching nuclei segmentation were then applied to find nucleus boundaries for accurate measurements. Finally, we performed positive grading analysis of NPC tissue sections for further clinical researches.

Comparing to traditional Ki-67 staining image segmentation methods, the improvements of our algorithm mainly included the following aspects. First, the unsupervised learning approach took pixel as the study object, overcame the inherent defect of low-quality image of the traditional method based on geometrical morphology, and no training samples were needed. Second, two color channels including B and H were selected from multiple color spaces. Features from those two channels enhanced the between-classes distances rather than other color expressions. Third, local correlation features were included with single pixel intensities, which improved accuracy of pixel clustering in consideration of neighborhood pixels in local area. Then, Watershed based on chessboard distance devided practical boundaries between overlapped nuclei, and finally, various indexes representing the positive grading of NPC tissue sections were generated for comprehensive analysis.

Meanwhile, the proposed algorithm converted segmentation issue into classification issue, which simplified the calculation and improved the efficiency of algorithm implementation. With further optimizations, for example, parallelized optimization, high speed and accuracy of this algorithm can be fully competent to meet the real-time requirements in clinical use, which may conduct preliminary screening for pathological experts to saves manpower and avoids subjective errors.

Positive grading is only one of the indicators in NPC tumor proliferative activity study. More morphological features characterizing status of individual cells could be derived from image segmentation results. A set of statistics could be derived from segmentation results such as nuclei size and shape quantitative factors. Further researches based on the morphological analysis of individual cell could be adopted to discover the relationship between cell microstructure and its activities.

The performance of Ki-67 staining image analysis was highly determined by nuclei labeling and touching nuclei segmentation methods. As discussed above, three types of error could be further avoided by improving these two core algorithms. Within the image processing pipeline, both kmeans clustering and Watershed segmentation could be replaced by other algorithms with more efficiency. The more precise boundaries of individual cells detected, the better view of cell activity study could be obtained.

By processing images and generating effective indicators in NPC tissue pathological analysis, this study can also been applied to microscopic images acquired from other staining techniques. Based on the robustness of clustering image segmentation methods, further improvements may focus on enriching detection indicators of tumor microstructure and increasing the segmentation accuracy of low quality stained tissues. The pipeline will be further optimized and integrated into a web released software for public use. The extension of proposed framework will be helpful for both NPC related pathological study and analysis of tissue section images acquired from other staining techniques.

## Additional Information

**How to cite this article**: Shi, P. *et al.* Automated Ki-67 Quantification of Immunohistochemical Staining Image of Human Nasopharyngeal Carcinoma Xenografts. *Sci. Rep.* **6**, 32127; doi: 10.1038/srep32127 (2016).

## Figures and Tables

**Table 1:** Values in RGB and HSV channels of brown and blue colors.

Values	RGB Space			HSV Space		
	R	G	B	H	S	V
Brown	150	75	0	0	75	65
Blue	0	0	255	240	100	100

**Table 2:** Main features and nuclei labeling performances of two automatic methods.

	ImmunoRatio	Our method
Object images	Ki-67, ER, and PR	Ki-67, HE
Background correction	Yes	Unnecessary
Color features	RGB	RGB and HSV
Stain separation	Color deconvolution based	Machine learning based
Training samples	No	No
Overlapping segmentation	Watershed	Watershed
DAB/nuclear number (positive percentage)	77.9 ± 7.1%	75.1 ± 6.7%
Error rate against manual labeling	9.4 ± 1.05%	8.2 ± 0.86%

**Table 3:** Positive grading quantification of 20 NPC tissue sections.

Quantification	Manual	Our method
DAB coloring strength (I_b_ value)	N/A	43.7 ± 1.9
DAB/nuclear number (positive percentage)	71.4 ± 5.5%	75.1 ± 6.7%
Positive grading	(+++) 14	(+++) 14
	(++) 5	(++) 5
	(+) 1	(+) 1
	(−) 0	(−) 0

## References

[b1] Pan J. *et al.* Early changes in apparent diffusion coefficients predict radiosensitivity of human nasopharyngeal carcinoma xenografts. Laryngoscope. 122, 839–843 (2012). 22374860 10.1002/lary.23208

[b2] Scholzen T. & Gerdes J. The Ki-67 protein: from the known and the unknown. J. Cell Physiol. 182, 311–322 (2000). 10653597 10.1002/(SICI)1097-4652(200003)182:3<311::AID-JCP1>3.0.CO;2-9

[b3] Madden S. F. *et al.* Breast mark: an integrated approach to mining publicly available transcriptomic datasets relating to breast cancer outcome. Breast Cancer Res. 15, R52 (2013). 23820017 10.1186/bcr3444 PMC3978487

[b4] Peng Y., Wang L. & Gu J. Elevated preoperative carcinoembryonic antigen (CEA) and Ki67 is predictor of decreased survival in IIA stage colon cancer. World J Surg. 37, 208–213 (2013). 23052808 10.1007/s00268-012-1814-7

[b5] van der Loos C. M. Multiple immunoenzyme staining: methods and visualizations for the observation with spectral imaging. J Histochem Cytochem. 56, 313–328 (2008). 18158282 10.1369/jhc.2007.950170 PMC2326109

[b6] Zehntner S. P., Chakravarty M. M., Bolovan R. J., Chan C. & Bedell B. J. Synergistic tissue counterstaining and image segmentation techniques for accurate, quantitative immunohistochemistry. J Histochem Cytochem. 56, 873–880 (2008). 18574255 10.1369/jhc.2008.950345 PMC2544616

[b7] Ranefall P., Wester K. & Bengtsson E. Automatic quantification of immunohistochemically stained cell nuclei using unsupervised image analysis. Anal Cell Pathol. 16, 29–43 (1998). 9584898 10.1155/1998/608293 PMC4617571

[b8] Loukas C. G., Wilson G. D., Vojnovic B. & Linney A. An image analysis-based approach for automated counting of cancer cell nuclei in tissue sections. Cytometry A 55, 30–42 (2003). 12938186 10.1002/cyto.a.10060

[b9] Gertych A., Joseph A. O., Walts A. E. & Bose S. Automated detection of dual p16/Ki67 nuclear immunoreactivity in liquid-based pap tests for improved cervical cancer risk stratification. Ann Biomed Eng. 40, 1192–204 (2012). 22215277 10.1007/s10439-011-0498-8 PMC3336006

[b10] Xing F., Su H., Neltner J. & Yang L. Automatic ki-67 counting using robust cell detection and online dictionary learning. IEEE Trans Biomed Eng. 61, 859–870 (2014). 24557687 10.1109/TBME.2013.2291703

[b11] Grala B. *et al.* New automated image analysis method for the assessment of Ki-67 labeling index in meningiomas. Folia Histochem Cytobiol. 47, 587–586 (2010). 20430724 10.2478/v10042-008-0098-0

[b12] Di Cataldo S., Ficarra E., Acquaviva A. & Macii E. Automated segmentation of tissue images for computerized IHC analysis. Comput Methods Programs Biomed. 100, 1–5 (2010). 20359767 10.1016/j.cmpb.2010.02.002

[b13] Schneider C. A., Rasband W. S. & Eliceiri K. W. NIH Image to ImageJ: 25 years of image analysis. Nat Methods. 9, 671–675 (2012). 22930834 10.1038/nmeth.2089 PMC5554542

[b14] Ruifrok A. C. & Johnston D. A. Quantification of histochemical staining by color deconvolution. Anal Quant Cytol Histol. 23, 291–299 (2001). 11531144

[b15] Tuominen V. J., Ruotoistenmaki S., Viitanen A., Jumppanen M. & Isola J. ImmunoRatio: a publicly available web application for quantitative image analysis of estrogen receptor (ER), progesterone receptor (PR), and Ki-67. Breast Cancer Res. 12, R56 (2010). 20663194 10.1186/bcr2615 PMC2949645

[b16] van Vliet L. J., Young L. T. & Verbeek P. W. Recursive gaussian derivative filters.*, 1998. proc. of Fourteenth International Conference on Pattern Recognition*, Brisbane, Qld. **1**, 509–514 (1998).

[b17] Sun T. N. & Neurvo Y. Detail-preserving median based filters in image processing. Pattern Recognit Lett. 15, 341–347 (1994).

[b18] Al-Lahham H. Z., Alomari R. S., Hiary H. & Chaudhary V. Automating proliferation rate estimation from Ki-67 histology images. proc. of SPIE Medical Imaging, International Society for Optics and Photonics. 83152A (2012).

[b19] Malpica N. *et al.* Applying watershed algorithms to the segmentation of clustered nuclei. Cytometry. 28, 289–297 (1997). 9266748 10.1002/(sici)1097-0320(19970801)28:4<289::aid-cyto3>3.0.co;2-7

[b20] Karvelis P. S., Tzallas A. T., Fotiadis D. I. & Georgiou I. A multichannel watershed-based segmentation method for multispectral chromosome classification. IEEE Trans Med Imaging. 27, 697–708 (2008). 18450542 10.1109/TMI.2008.916962

[b21] Vincent L. & Soille P. Watersheds in digital spaces: an efficient algorithm based on immersion simulations. IEEE Trans Pattern Anal Mach Intell. 13, 583–598 (1991).

[b22] Berry N. *et al.* The prognostic value of the monoclonal antibodies HMFG1 and HMFG2 in breast cancer. Br J Cancer. 51, 179 (1985). 2578285 10.1038/bjc.1985.27 PMC1977039

[b23] Fromowitz F. B. *et al.* RAS p21 expression in the progression of breast cancer. Hum Pathol. 18, 1268–1275 (1987). 3315956 10.1016/s0046-8177(87)80412-4

